# Interaction torque contributes to planar reaching at slow speed

**DOI:** 10.1186/1475-925X-7-27

**Published:** 2008-10-22

**Authors:** Hiroshi Yamasaki, Yoshiyuki Tagami, Hiroyuki Fujisawa, Fumihiko Hoshi, Hiroshi Nagasaki

**Affiliations:** 1 Department of Physical Therapeutics, School of Nursing and Rehabilitation, Showa University, 1865 Toka-ichiba, Midori-ku, Yokohama, 226-8555, Japan; 2 Department of Rehabilitation, Faculty of Medical Science and Welfare, Tohoku Bunka Gakuen University, Sendai, Japan; 3 School of Health and Social Service, Saitama Prefectural University, Saitama, Japan

## Abstract

**Background:**

How the central nervous system (CNS) organizes the joint dynamics for multi-joint movement is a complex problem, because of the passive interaction among segmental movements. Previous studies have demonstrated that the CNS predictively compensates for interaction torque (INT) which is arising from the movement of the adjacent joints. However, most of these studies have mainly examined quick movements, presumably because the current belief is that the effects of INT are not significant at slow speeds. The functional contribution of INT for multijoint movements performed in various speeds is still unclear. The purpose of this study was to examine the contribution of INT to a planer reaching in a wide range of motion speeds for healthy subjects.

**Methods:**

Subjects performed reaching movements toward five targets under three different speed conditions. Joint position data were recorded using a 3-D motion analysis device (50 Hz). Torque components, muscle torque (MUS), interaction torque (INT), gravity torque (G), and net torque (NET) were calculated by solving the dynamic equations for the shoulder and elbow. NET at a joint which produces the joint kinematics will be an algebraic sum of torque components;

NET = MUS - G - INT.

Dynamic muscle torque (DMUS = MUS-G) was also calculated. Contributions of INT impulse and DMUS impulse to NET impulse were examined.

**Results:**

The relative contribution of INT to NET was not dependent on speed for both joints at every target. INT was additive (same direction) to DMUS at the shoulder joint, while in the elbow DMUS counteracted (opposed to) INT. The trajectory of reach was linear and two-joint movements were coordinated with a specific combination at each target, regardless of motion speed. However, DMUS at the elbow was opposed to the direction of elbow movement, and its magnitude varied from trial to trial in order to compensate for the variability of INT.

**Conclusion:**

Interaction torque was important at slow speeds. Muscle torques at the two joints were not directly related to each other to produce coordinated joint movement during a reach. These results support Bernstein's idea that coordinated movement is not completely determined by motor command in multi-joint motion. Based on the data presented in this study and the work of others, a model for the connection between joint torques (muscle and passive torques including interaction torque) and joint coordination is proposed.

## Background

Unlike the single joint movement, the dynamics of multi-joint movement is complex. Specifically, interaction torque (INT) will be included into the joint dynamics, which is arising from the movement of the adjacent joints [1]. INT at a joint, therefore, may be regarded by the CNS as an unavoidable passive disturbance which is to be adjusted for multijoint movement coordination.

Except for movement in the horizontal plane, net torque (NET) produces joint kinematics that can be shown as an algebraic sum of muscle (MUS), interaction (INT), and gravity torque (G);

(1) NET = MUS - INT - G

Previous studies on healthy subjects have shown that INT arising during rapid multi-joint movement is of sufficient magnitude to influence movement trajectory [1,2]. These authors have postulated that the CNS can predictively compensate or utilize INT [3-7]. INT in multi-joint dynamics have been investigated mainly for reaching movements [8-13]. Studies on patients with cerebellar lesions [14-17] or without proprioception [18-20] have an inability to control interaction torques, thereby resulting in kinematic deficits.

INT is dependent on joint acceleration and velocity. Motion speed has thus been considered as an essential factor for the effects of INT on multi-joint movement. However, it was pointed out qualitatively that the relative contribution of velocity was significant regardless of the movement speed [1]. Functional role of the interaction and muscle torque for the production of planar reaching through different movement speed is still unclear.

The primary purpose of this study was to re-examine whether the effects of interaction torque on multi-joint reaching movement is significant at slow speed. Planar reach involving shoulder and elbow joints toward different targets in the sagittal plane was examined under a wide range of motion speeds.

It is known that the trajectory of reach and joint coordination have invariant characteristics irrespective of target position and movement speed [21]. If the effect of the interaction torque was significant over a range of different speed, then a question arises as to how the relation of muscle torque between neighboring joints is adequately produced to achieve multi-joint coordination. Therefore, a secondary purpose of this study was to explore whether the coordination between muscle torques at two joints corresponds to coordinated joint movements.

## Methods

### Subjects

Ten right-handed healthy adults (five males and five females) gave an informed consent to participate in this study. All protocols were approved by the Review Board of Tohoku Bunka Gakuen University. They had no neurological, musculoskeletal, or visual disorders by self-report. The subject's age ranged from 20 to 22 years (average 21 yrs).

### Tasks

The subjects sat on a stool with their shoulder 0° flexed, 0° adducted and 0° outward rotated. Their right elbow joint and hand were flexed 90° and at a middle position to supination and pronation, respectively. The subjects were asked to reach their right hand by pointing at a small target in the sagittal plane while keeping their trunk in the initial position. Each target was 1 cm in width and wrapped around a stick of 1.5 cm diameter that stood in front of the right shoulder joint. The subjects were verbally instructed to reach at fast, natural (comfortable), and slow speed. Accuracy of the pointing was not required.

**Figure 1:**
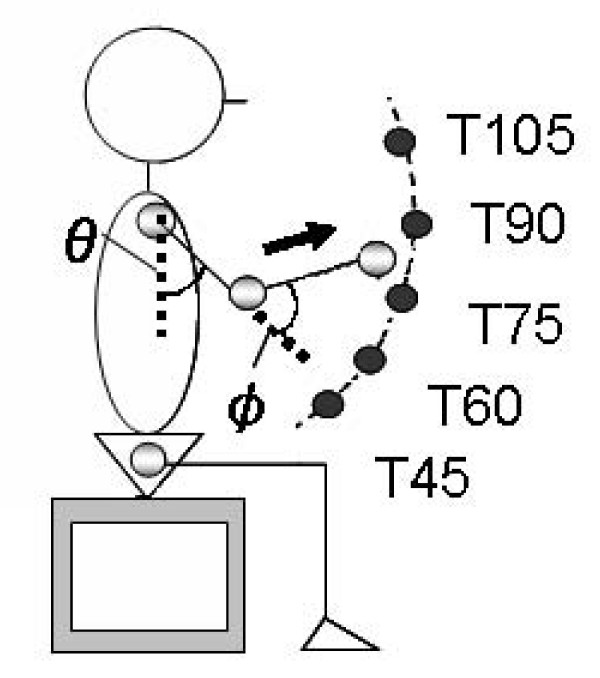
**Target position**. Five small targets (1.0 × 1.5 cm) were placed in front of the shoulder joint of the subject. The distance from the shoulder joint was adjusted to 80% of the upper arm length of the each subject. Target positions required shoulder flexion of 45 deg (T45), 60 deg (T60), 75 deg (T75), 90 deg (T90), and 105 deg (T105).

As shown in Figure. 1, we used five targets that required subjects to flex their shoulder from 45 to 105 degrees at 15 degrees intervals (i.e., 45 degrees (T45), 60 degrees (T60), 75 degrees (T75), 90 degrees (T90), and 105 degrees (T105)). The distance of the target from the shoulder was adjusted to 80% of their upper arm length.

Subjects were allowed to practice several times by reaching at T45 to familiarize themselves with the task. All subjects first reached to T60 under a slow speed condition, and then target and motion speed were randomly assigned. The subjects performed a total of 15 trails (5 targets × 3 speeds).

### Data collection and analysis

Adhesive infra-red reflex markers were placed on the acromion, the lateral epicondyle of the humerus, and middle point between styloid process of the ulnaris and radius of the right arm, and positions were collected using a three-dimension motion analysis device (ELITE puls, BTS, 50 Hz). Position data of each joint was smoothed with a cutoff frequency of 3 Hz for natural and slow speed conditions, and 1 Hz for the very slow speed condition using a second order Butterworth filter [22]. Using these position data, joint angle, velocity, and acceleration were calculated for the shoulder and elbow joints.

### Kinematics

#### Movement time

Since the angular velocity profile had always a single peak, peak angular velocity was calculated for each joint in each trial. The onset and termination of movement were determined at the time where the angular velocity of the shoulder or elbow exceeded 5% of its peak. The duration from the onset to the termination of movement was defined as movement time (MT).

#### Trajectory curvature index

Wrist path was quantified by curvature index (I = d/L) determined as the ratio of maximum path deviation (d) from a straight line (L) connecting the initial and final points of the wrist trajectory. A deviation under the straight line was evaluated as positive.

### Kinetics

#### Calculation of torque components

The dynamic equations for two-linked rigid bodies composed of the upper and lower arms were used to calculate the joint torque components (i.e., MUS, NET, INT, and G for the shoulder and elbow joint)(Additional file 1). Anthropometric data were estimated from the height and weight of each subject [23].

#### Absolute torque impulse

The magnitude of each torque component was quantified by calculating absolute torque impulse during movement time. The G at the joints is a function of the angle and load, and is independent of movement speed (see Additional file 1). Because this study aimed to examine dynamic changes in muscle torque components, we calculated the "dynamic muscle torque" [24]. Dynamic muscle torque (DMUS) was the residual muscle torque after removing the gravitational component. The impulse of DMUS was similarly calculated.

#### Contribution index

We quantified the relative contributions of DMUS and INT to NET as follows [9]. In a period during which the INT was in the same direction as NET, the INT impulse was evaluated as positive, while when the INT was in the opposite direction to NET, the INT impulse was evaluated as negative. The total sum of the positive and negative INT impulses over movement time was divided by the absolute impulse of NET to yield a contribution index of INT to NET in each trial. Similarly, the positive and negative impulses of the DMUS were summed to yield a contribution index of DMUS to NET. The sum of both indexes was always 1.

## Results

### Kinematics

#### Movement time (MT)

MT averaged across subjects is shown for each target in Table 1. MTs at the fast, natural, and slow conditions were around 800 ms, 1200 ms, and 2200 ms, respectively. This shows that the subjects performed the movements at "natural", "slow", and "very slow" speeds. Two-way ANOVA (speed × targets) found a significant effect of speed (F = 78.22, p < 0.01), but not target. This indicates that the subjects regulated their reaching velocity such that MT was kept constant regardless of the target position.

**Table 1:** Movement time [ms]

Target	Speed condition					
	Fast		Natural		Slow	
	
T45	766	(120)	1312	(441)	2260	(840)
T60	804	(113)	1300	(206)	2142	(1074)
T75	798	(113)	1212	(119)	2040	(473)
T90	882	(148)	1396	(249)	2240	(808)
T105	769	(135)	1424	(367)	2507	(955)

Mean (SD) for all subjects.

#### Trajectory

**Figure 2:**
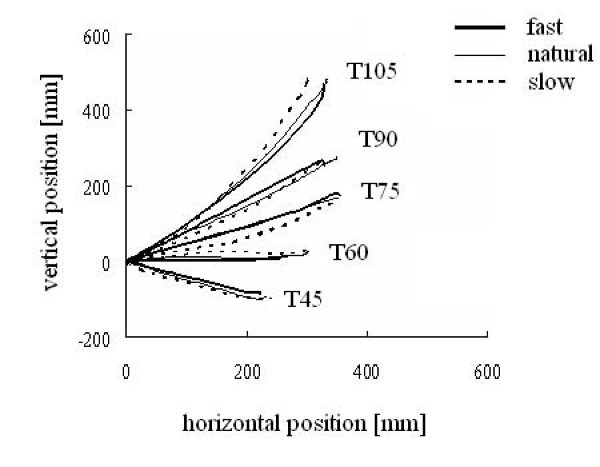
**Space coordination**. A typical example from one subject of wrist trajectories during reaching to all targets and speed conditions.

The wrist trajectory from a subject is superimposed for three movement speeds to each target in Figure 2. The path from the start position to the target was almost straight, regardless of movement speed (i.e., reaching was performed to trace the shortest path from start to the final position). This pattern was observed in all subjects. The curvature index of wrist trajectory ranged from -0.07 to 0.108. Two-way ANOVA showed significant main effect of the target on the curvature index (F = 15.21, p < 0.01), but not speed. Tukey HSD analysis found that the curvature index for T90 and T105 are significantly greater than the other three targets (p < 0.01). Also, t-test showed the curvature index for T90 and T105 are significantly different from zero in all speed conditions (p < 0.01), but the curvature index for T45, T60, and T75 in all speed conditions were not significantly different from zero. These results indicate that the wrist trajectories for T45, T60, and T75 are linear irrespective of the target position or speeds. This invariant wrist trajectory for lower three targets implies that the trajectory was constrained to be linear, thus diminishing a degree of freedom in the upper extremity [25].

#### Joint co-ordination

**Figure 3:**
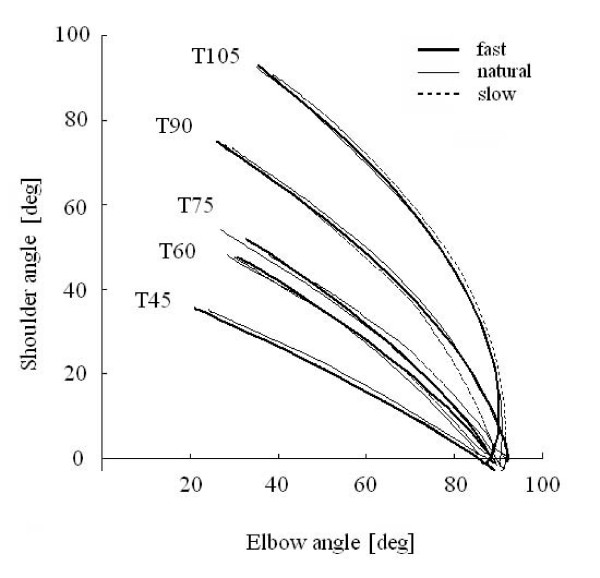
**Joint coordination**. Relationship between angular displacements of the elbow and shoulder during reaching to all targets and speed conditions from the same subject in Fig. 2.

Figure 3 shows the relationship between the angles of the shoulder and elbow for the same trials as shown in Figure 2. As seen with the wrist trajectory, the changes in shoulder and elbow angles were constrained to a specific combination at each target. Although the targets employed in this study required different shoulder and elbow excursions, a coordinated relationship between shoulder and elbow joints was observed irrespective of reaching speed. Since the model used in the present study is a non-redundant system, the angles of the joints are determined uniquely from an endpoint coordinate. The relationship between shoulder and elbow angles to a target were similar at different speeds. Note that while reaching to T105 the shoulder joint initially flexed slightly, and after a short delay, the elbow began to extend.

#### Joint angular velocity

**Figure 4:**
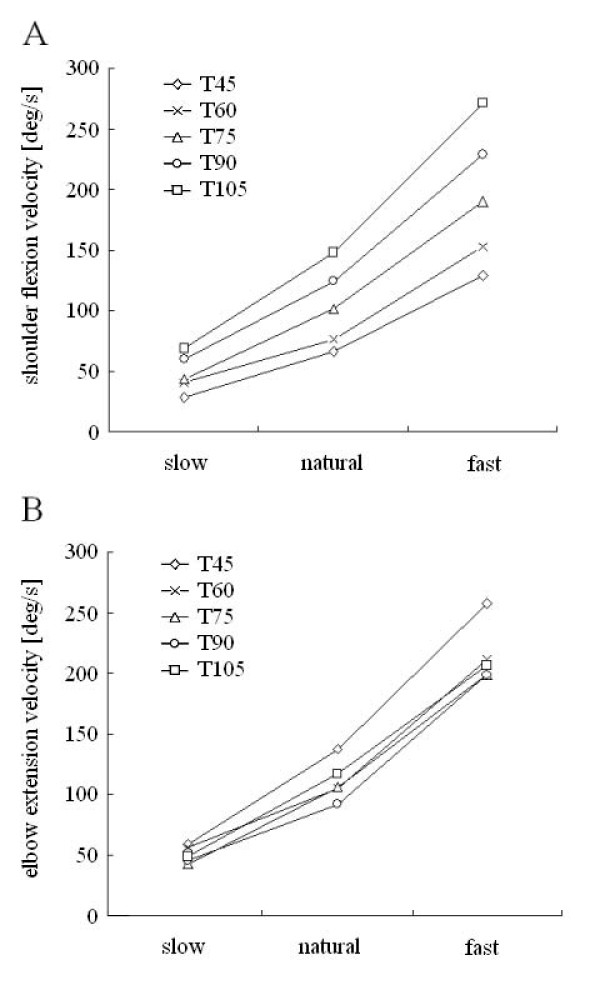
**Peak angular velocity (PV)**. A: PV of shoulder flexion against speed conditions. B: PV of elbow extension against speed conditions.

Peak angular velocity of the joints is shown for each target in Figure 4A and 4B. There was a significant effect of target (F = 58.01, p < 0.001 for the shoulder, F = 7.92, p < 0.001 for the elbow) and speed (F = 425.36, p < 0.001 for the shoulder, F = 375.94, p < 0.001 for the elbow). The interaction effect of target and speed was significant for the shoulder (F = 6.632, p < 0.001, Figure 4A). For the shoulder, examining the simple effect of interaction revealed the significant target effects at each speed (F = 49.43, p < 0.001 for fast, F = 17.98, p < 0.001 for natural, F = 3.85, p < 0.01 for slow), also the effects of speed were significant for all targets (F = 41.25, p < 0.001 for T1, F = 52.53, p < 0.001 for T2, F = 87.401, p < 0.001 for T3,, F = 115.09, p < 0.001 for T4,, F = 149.76, p < 0.001 for T5). Depending on the excursion of each joint, peak angular velocity of the shoulder joint increased, while the elbow velocity tended to decrease as the target became higher.

### Kinetics

#### Torque components

**Figure 5:**
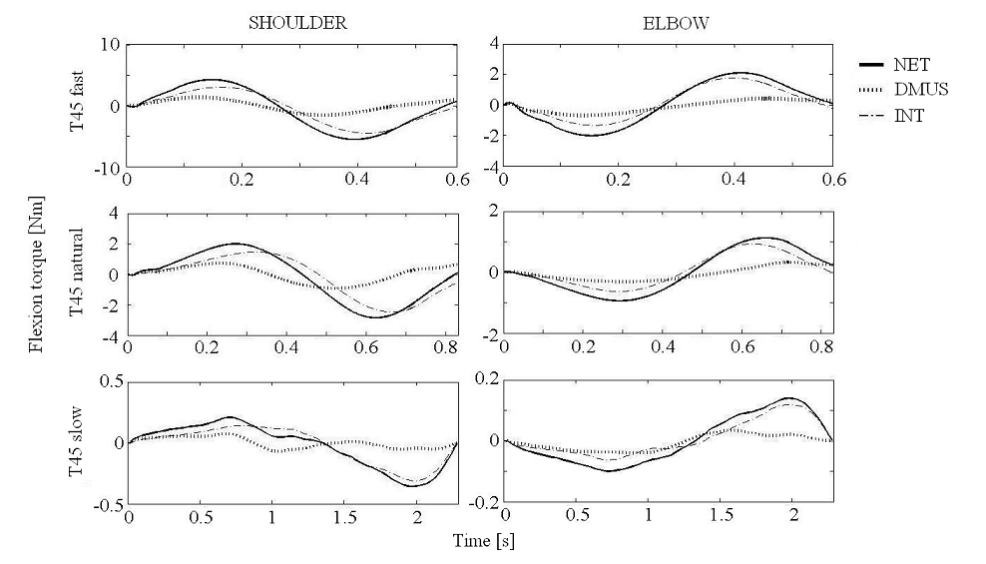
**Torque profile for T45 reaching**. Time course for the torque components during T45 reaching from one subject. Positive values in ordinate indicates flexion. NET: net torque, INT: interaction torque. DMUS (dynamic muscle torque) is shown instead of muscle and gravity torque.

**Figure 6:**
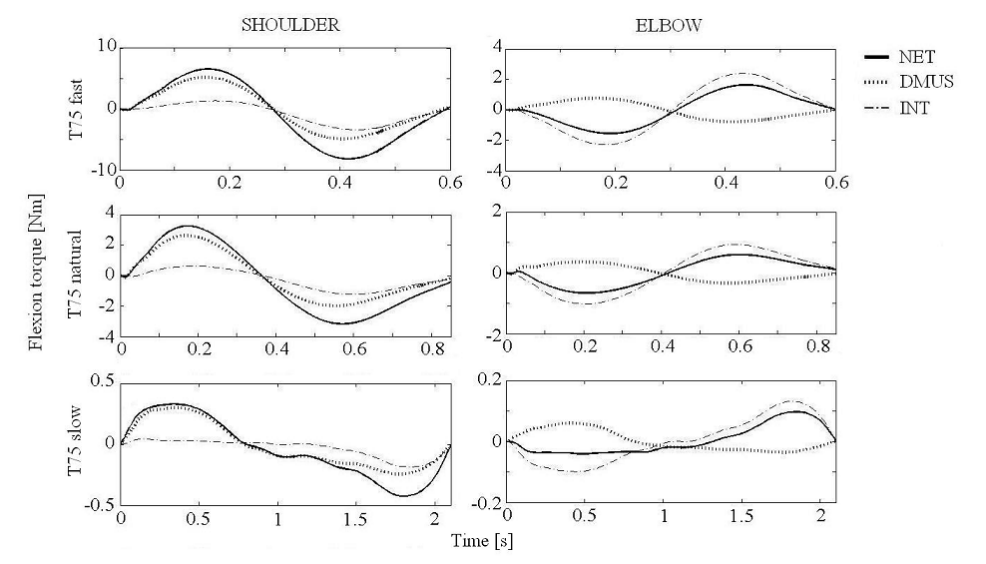
**Torque profile for T75 reaching**. Time course for the torque components during T75 reaching from one subject. Positive values in ordinate indicates flexion. NET: net torque, INT: interaction torque. DMUS (dynamic muscle torque) is shown instead of muscle and gravity torque.

**Figure 7:**
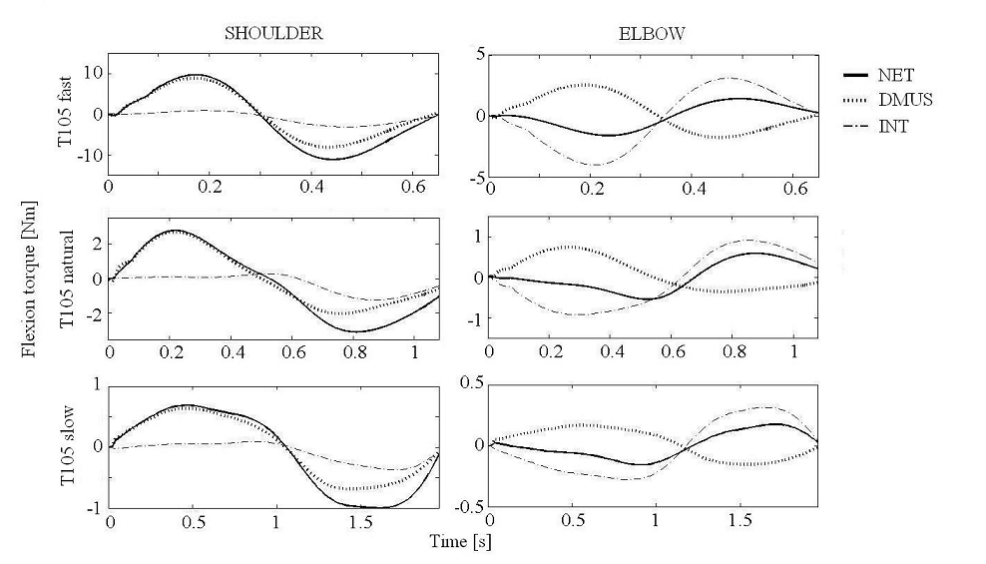
**Torque profile for T105 reaching**. Time course for the torque components during T105 reaching from one subject. Positive values in ordinate indicates flexion. NET: net torque, INT: interaction torque. DMUS (dynamic muscle torque) is shown instead of muscle and gravity torque.

Representative torque profiles in both joints of a subject are shown in Figures 5, 6, 7 for three speed conditions, to target T45 (Figure 5), T75 (Figure 6) and T105 (Figure 7). NET and INT appeared to be a sinusoidal wave form with the same direction and phase in each joint. All subjects showed similar patterns. At the shoulder, flexion dynamic muscle torque (DMUS) changed roughly in phase with NET, indicating DMUS contributed to NET in the same direction. In contrast, DMUS at the elbow showed an anti-phase pattern against NET as shown Figure 6 and Figure 7, indicating DMUS contributed to NET in the opposite direction for T75 and T105. Note that the first peak of elbow DMUS occurred prior to that of the NET in reaching to T105.

Although NET and interaction torque (INT) were small at slow speed, the relation between DMUS and NET was preserved for all speed conditions at both joints. That is, DMUS utilized (in the shoulder) or compensated (in the elbow) INT in order to generate the required joint movement.

#### Torque impulses

**Figure 8:**
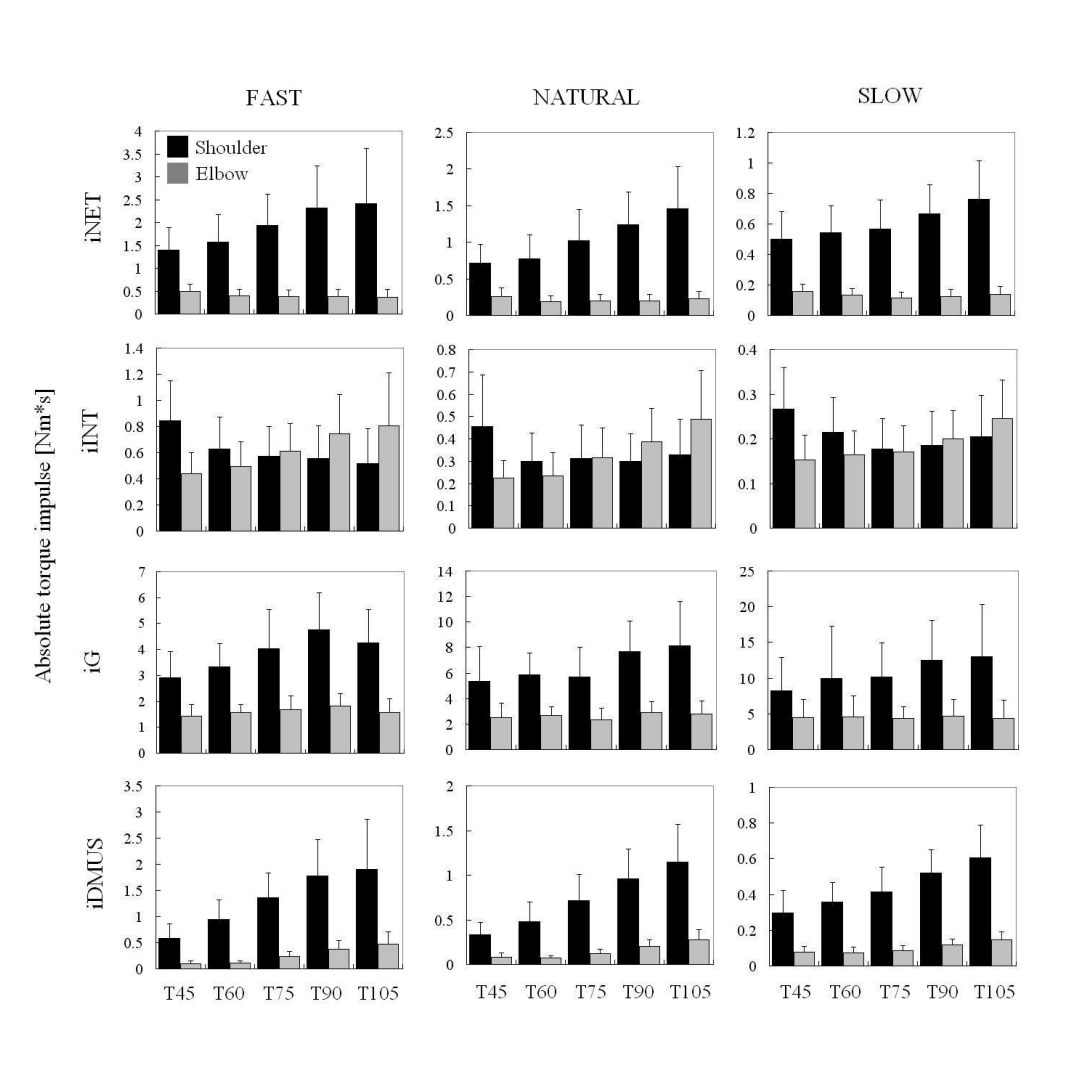
**Absolute torque impulse**. Averaged absolute torque impulses against the target height at each joint under different speed conditions. iNET: absolute impulse of the net torque, iINT: absolute impulse of the interaction torque, iG: absolute impulse of the gravity torque, iDMUS: absolute impulse of the dynamic muscle torque.

Figure 8 shows the absolute torque impulses for each target at each joint under different speed conditions. The magnitude of the impulses was affected by the movement speed and target. The absolute NET impulse (iNET) of the shoulder increased consistently as the target elevated (F = 10.91, p < 0.01), while iNET of the elbow tended to decrease (F = 2.00, p = 0.09). These changes corresponded to the changes in peak angular velocity of both joints (Figure 4). The absolute INT impulses (iINT) of the shoulder decreased with the target height (F = 4.86, p < 0.01). In contrast, iINT of the elbow increased with the target (F = 12.39, p < 0.01). Note that magnitudes of iINT of both joints were comparable, while iNET of the shoulder was far greater than iNET of the elbow under all conditions. Consequently, the contribution of INT to NET became more dominant at the elbow than the shoulder. Although the magnitude of gravity torque impulse (iG) of the shoulder increased with target height (F = 4.00, p < 0.01), iG of the elbow was independent of this variable. The magnitude of iG at slow speed was far greater than at fast speed because of a longer movement time. The DMUS impulse (iDMUS) of both joints increased with the target height (F = 27.90, p < 0.01 for the shoulder, F = 42.11, p < 0.01 for the elbow). This indicates that neither the speed nor distance solely determined the magnitude of iDMUS. At the shoulder, the sum of iINT and iDMUS was approximately equal to iNET for each target, but this was not the case at the elbow.

#### Relative contribution of muscle and interaction torques to NET torque

**Figure 9:**
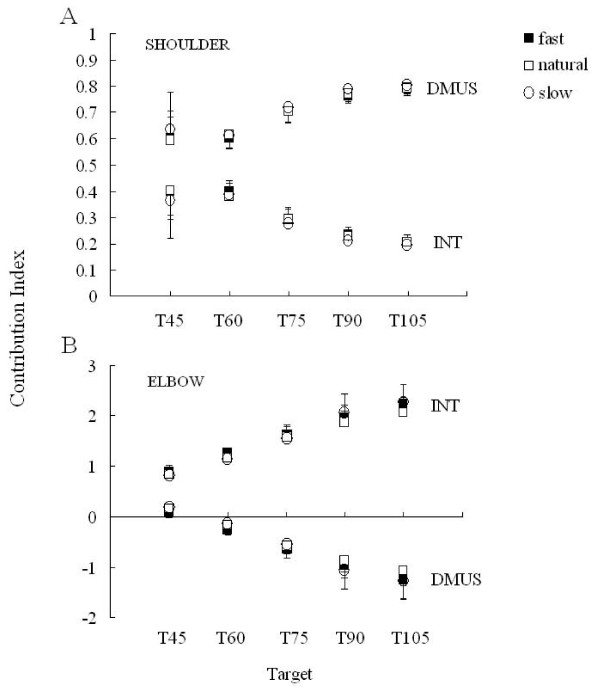
**Contribution index**. Averaged relative contributions of the interaction torque (INT) and the dynamic muscle torque (DMUS) to the net joint torque (NET) against the target in both joints.

The relative contributions of INT and DMUS to NET (i.e., the contribution index defined above in the methods section), exhibited systematic changes with the target in both joints. As shown in Figure 9A, DMUS became a main contributor to NET as the target got higher at the shoulder. Also, the contribution index of INT and DMUS were always positive and remained below 1 (always less than NET), indicating that the two components additively contributed to NET. The target had a significant effect on DMUS (F = 209.54, p < 0.01) and INT (F = 209.23, p < 0.01), but not with the speed condition.

The contribution index for elbow INT changed with the target (F = 167.17, p < 0.01) and was always positive and greater than 1, while the DMUS contribution index changed to negative with target height (F = 166.56, p < 0.01) (Figure 9B). Similar to the shoulder, there was no significant effect on the speed condition. The positive contribution of DMUS was found only in T45. In order to produce NET, there was a counteractive relationship between DMUS and INT at the elbow (i.e., the excess INT had to be counteracted by DMUS in order to generate the required NET). Interestingly, the DMUS contribution exhibited almost zero at T45 or T60, regardless of the movement speed. Specifically, the muscle torque was used only to cancel the gravity. In sum, the relative contribution of INT and DMUS to NET was not dependent on reaching speed at every target, both for the shoulder and elbow joints.

### Coordination between torque components and kinematics

**Figure 10:**
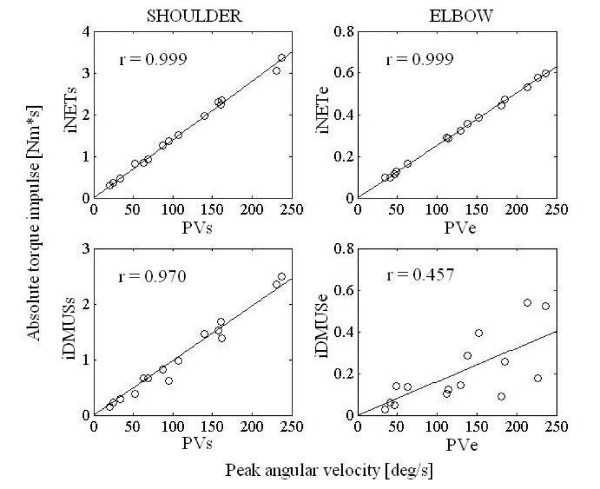
**Relation between the magnitude of torque impulses and peak angular velocity**. iNET: Absolute impulse of the net torque, iDMUS: Absolute impulse of the dynamic muscle torque, PV: Peak angular velocity. Lowercase letter "s": shoulder, "e": elbow.

As described earlier, there was an invariant relationship in trajectory (Figure 2) and joint movement (Figure 3). Figure 10 illustrates the relationship between the magnitude of torque impulses and peak angular velocity for all targets from one subject. As expected, a high correlation between iNET and peak angular velocity were observed in each joint (r = 0.999, p < 0.01 for both joints). The correlations between iDMUS and peak angular velocity were also high for the shoulder (r = 0.970 p < 0.01), but the correlation was greatly reduced in the elbow (r = 0.457(n.s.)). The correlation coefficients averaged across all subjects are shown in Table 2. Table 2 demonstrates that iDMUS in the elbow, in contrast to the shoulder joint, had only a weak correlation with peak elbow velocity as compared to other torque components. The correlation of torque impulse to peak angular velocity at the elbow joint was held in terms of NET but not in DMUS. Note that the direction of DMUS was always opposed to the movement direction, except at T45.

**Table 2:** Correlate coefficients between peak angular velocity and torque impulse.

Subject	PV s – iNETs	PV e – iINTs	PV s – iDMUSs	PV e – iNETe	PV s – iINTe	PV e – iDMUSe
1	0.998 **	0.986 **	0.989 **	0.999 **	0.998 **	0.720 **
2	0.999 **	0.993 **	0.985 **	0.999 **	0.990 **	0.484 n.s
3	0.994 **	0.985 **	0.965 **	0.997 **	0.990 **	0.394 n.s
4	0.998 **	0.979 **	0.981 **	0.998 **	0.998 **	0.550 *
5	0.998 **	0.991 **	0.983 **	0.999 **	0.999 **	0.557 **
6	0.996 **	0.979 **	0.964 **	0.998 **	0.994 **	0.173 n.s
7	0.999 **	0.991 **	0.980 **	0.998 **	0.997 **	0.589 *
8	0.996 **	0.962 **	0.976 **	0.996 **	0.996 **	0.494 n.s
9	0.999 **	0.987 **	0.971 **	0.999 **	0.998 **	0.341 n.s
10	0.999 **	0.988 **	0.970 **	0.999 **	0.995 **	0.457 n.s

PV: Peak angular velocity, s: for the shoulder, e: for the elbow. iNET: Absolute torque impulse of net torque, iINT: Absolute torque impulse of interaction torque, iDMUS: Absolute impulse of dynamic muscle torque. ** p < 0.01, * p < 0.05

## Discussion

### Kinematic invariance of reach and kinetic contributions

The experimental setting adopted in the present study was designed to simulate every day reaching in which subjects performed forward reaching in a vertical plane. Reaching targets differed both in direction and distance. The distance increased as the target got higher from T45 to T105 (Figure 2), but movement time remained the constant among targets (Table 1) because of a corresponding increase in the velocity of reach. Since increases in shoulder velocity with higher targets were compensated by decreases in elbow velocity (Figure 4), movement time was kept constant independent of the target position. The changes in angular velocity were directly related to the amount of net torque (iNET) required for reaching in the shoulder and elbow (Figure 8).

In addition, differences in target direction corresponded to gravity torque loaded on the both joints. Whereas gravity torque on the shoulder joint increased steadily with the target height, gravity on the elbow changed little (Figure 8). The effect of gravity on the trajectory generation has been examined by some researchers. A series of studies by Papaxanthis *et al*. showed that the direction of pointing movement was a determinant for arm trajectory generation in a vertical plane [26-29]. The result of the present study is in agreement with their result, that is, the linearity of hand trajectory varied with the direction of reach. In particular, the trajectories of reaching movement to upper targets in this study, i.e., T90 and T105, were curved more greatly than the lower targets. This result implies that gravity affects the trajectory in vertical reaching.

Further, the present study suggests gravity and interaction torque both relate to wrist trajectory generation in multijoint movement. If the absolute magnitude of interaction torque was a major factor for trajectory generation, changes in the linearity of wrist trajectory would appear under different speed conditions. While the magnitude of the interaction torque (iINT) decreased as the reach got slower (Figure 8), the linearity of trajectory to a target was independent of the speed. Because of the speed-independency of the trajectory, it is suggested that the interaction torque contributed to the trajectory formation even at slow speed. This point will be discussed below.

### Speed is independent of the relative contribution of interaction torque to net torque

We believe that the most remarkable finding of this study was that the contribution of INT to NET was important when reaching at slow speed. The contribution index of INT to NET was independent of speed for every target in the shoulder and elbow joints (Figure 9). As the velocity of reach decreases, the magnitude of INT and NET at the joints also decrease. This relation might be the reason for the fact that the relative contribution of INT to NET was independent of the speed. A recent study from our laboratory examined squatting and also showed the same speed-independency of the relative contribution of INT to NET (manuscript in preparation). It has been postulated that the speed of reaching is crucial for the effects of interaction torque, and therefore may be negligible for multi-joint movements at slow speed [30]. Consequently, most recent studies have examined the effects of INT on fast movements.

Messier et al. [20] reported that patients with complete loss of proprioception experience a deficit in accuracy during slow reaching, and also suggested that the proprioceptive information providing cues for the predictive control of interaction torque is important, even when interaction torques are very small. Also, Hollerbach and Flash [1] demonstrated qualitatively that the effects of interaction torque may be relatively independent of speed. Our result is consistent with these works and suggests that the contribution of interaction torque to multi-joint movement may be significant irrespective of speed.

### Direction-specific interaction torque

The contribution index of INT consistently depended on the target direction at both joints. This result is consistent with data presented in previous studies demonstrating that the magnitude of INT gradually changes with the direction of reaching [9,10,12]. As a larger shoulder flexion was required for the elbow extension (e.g., reaching toward T105), the contribution of iDMUS became more dominant than that of INT at the shoulder, while the INT contributed excessively to NET at the elbow. In contrast, when reaching toward T45 the contribution of INT became dominant when compared to iDMUS at the shoulder. The dependence of INT on the target position shown in our study could be attributed to changes in the joint angular velocities of the shoulder and elbow joints. For instance, the peak angular velocity at the shoulder during reaching toward T105 was faster than the others (see Figure 4), thus resulted in more interaction torque produced at the elbow (Figure 8).

### The role of interaction torque and dynamic muscle torque

For every target, flexion DMUS and flexion INT always contributed in an additive fashion to flexion movement at the shoulder (i.e., INT assisted the shoulder movement). In other words, DMUS utilized the INT to produce a specific shoulder movement over a range of movement speeds. This can especially be seen by the contribution of DMUS to joint movement during T105 reaching. On the other hand, the contribution of DMUS also counteracted elbow movement. Because the INT overwhelmingly contributed to net torque production, DMUS was forced to compensate for the magnitude of the INT. Interestingly, when reaching to T45 and T60 the contribution of the DMUS to NET was quite low and the joint motion was produced solely by passive interaction torques caused by the shoulder joint.

Our results were consistent with the "shoulder-centered pattern" [12] or "leading joint hypothesis" [30], in which shoulder muscle torque predominates in the production of movement while the elbow muscles play a minor role. The torque profiles at the elbow (Figure 7) showed that DMUS was generated prior to NET. This result is consistent with the notion that the central nervous system (CNS) can predict coming interaction torque during multi-joint movement in a feed-forward manner [3-6,8,31].

### Implications for multi-joint coordination

The trajectory of reach examined in the present study was always linear to each target and the linearity was preserved regardless of movement speed (Figure 2). The linear trajectory of reach has been generally observed in planar reaching, except in the extreme margins of the work space [21]. Therefore, the angular movements of joints involved in reaching should be also coordinated so that a linear trajectory is produced. This inter-joint coordination is demonstrated in Figure 3 for our reaching task. The relationship between shoulder and elbow joints is the result of inverse kinematics from the linear trajectory.

The question then arises as to how muscular torque acts on each joint in order to produce coordinated joint movement, or "By what rule has the CNS selected an appropriate muscle activation pattern to achieve the inter-joint coordination?" [2]. If interaction torque can be neglected in multi-joint reaching, then the inter-joint coordination should closely correspond to the invariant relationship between the muscle torques of each joint. However, the joint net torque that produces inter-joint coordination cannot be determined solely by muscle torque (or dynamic muscle torque), because of the essential contribution of INT to NET. Consequently, muscle torques per se may be variable from trial to trial without corresponding to joint kinematics. This indeterminacy of muscle torque is indeed demonstrated in Figure 10 and Table 2 for the elbow joint in our reaching task. Also, the dynamic muscle torque could not predict the elbow peak velocity. In addition, the elbow muscle torque was opposed to the direction of the elbow movement.

Gottlieb *et al*. [32,33] previously reported data indicating that shoulder and elbow torques keep a linear relation in averaged trials of their reaching task (i.e., "linear synergy"). Based on this data, Gottlieb *et al*. postulated that the CNS uses a single command that is transmitted to the muscle at two joints in a predetermined proportion, thereby reducing the degree of freedom for movement. However, their later work failed to provide evidence that shows the generality of "linear synergy" for reaching [24]. Their studies on elbow muscle torque appeared to show linear relationship with shoulder torque, but it was often opposed to the direction of elbow movement depending on the target direction. The central command must designate the direction of elbow torque in this case, thereby requiring an additional degree of freedom.

**Figure 11:**
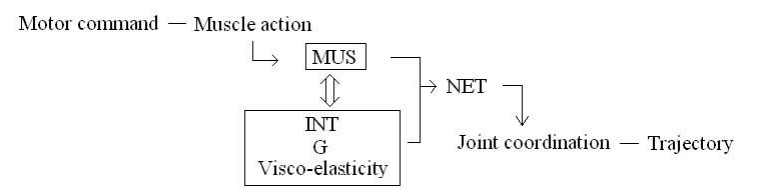
**Non-deterministic connection between muscle activation and inter-joint coordination**. MUS: muscle torque, INT: interaction torque, G: gravity torque, NET: net torque.

The indeterminacy of motor command to produce inter-joint coordination has been previously suggested [34] (i.e., "functional non-univocality of the connections between the motor center and the periphery"). Bernstein stressed that "movements are not completely determined by effector process" (P105). Consistent with this idea, the findings in this study suggest that the traditional notion of deterministic connection from motor command (and hence, muscle activation) to the coordinated movement is no longer holds true for multi-joint movements. Alternatively, our data indicate that motor command must adjust the direction and magnitude of dynamic muscle torque to passive torques on a trial to trial basis so that the coordinated joint movements are organized. A possible connection between muscle and passive torque, and the joint coordination in multi-joint movement, is illustrated in Figure 11. The passive torques in Figure 11 include interaction and gravity torques, and also the torque due to visco-elastic forces within muscle tissue [35]. Of particular interest is the behavior of elbow muscle torque examined in this study. Dynamic muscle torque at the elbow tended towards an opposite direction of elbow movement (Figure 9), and its magnitude varied from trial to trial in order to compensate for the variability of interaction torques at the joint (Figure 10). Nevertheless, the joint coordination was kept invariant irrespective of motion speed. We speculate that motor command does not control muscle activity by a rigid computational rule, and the CNS may instead have to learn through everyday experience to adjust muscle torque production against the perturbation caused by these passive torques.

## Conclusion

The relative contributions of interaction torque to net torque were independent of reaching speed. Effect of the interaction torque was significant in reaching movement even at slow speeds. Muscle torque at the two joints was not adequately related to each other to produce coordinated joint movement. These results support Bernstein's idea on the multi-joint coordination.

## Competing interests

The authors declare that they have no competing interests.

## Authors' contributions

HY carried out the construction of analysis environment, analyzed the data, and drafted the manuscript. YT participated in the data acquisition, analyzed the kinematic data, and drafted the manuscript. HF checked the mathematical procedure and carried out the kinetic analysis. FH participated in data analysis process. HN organized the study and helped to draft the manuscript. All authors read and approved the final manuscript.

## Supplementary Material

Additional file 1 Appendix. This is the appendix for the manuscript describing the definition of the torque components. Click here for file
