# Supplementary material for: Interaction torque contributes to planar reaching at slow speed
Source: Biomed Eng Online. 2008 Oct 22;7:27. doi: 10.1186/1475-925X-7-27 (PMC2577090; doi:10.1186/1475-925X-7-27)
Supplement: Additional file 1 — Appendix. This is the appendix for the manuscript describing the definition of the torque components. [file 1475-925X-7-27-S1.doc]

# Appendix

We used a dynamic equation for a two-linked rigid model to calculate the joint torque components. We define the “muscle torque (MUS)” as the net torque due to all muscles and passive tissue contributed to a joint movement, and the “net torque (NET)” as the sum of all torque components (muscle, interaction (INT), and gravitational (G) torque).

## Shoulder

NETs＝{m_1_l_1_^2^+m_2_(L_1_^2^+l_2_^2^+2L_1_l_2_cosφ)+I_1_+I_2_}$\ddot{\theta}$

INTs={m_2_l_2_(l_2_+L_1_cosφ)+I_2_}$\ddot{\varphi}$-(m_2_L_1_l_2_sinφ) $\dot{\varphi}^{2}$-2m_2_L_1_l_2_ sinφ

Gs=(m_1_l_1_+m_2_L_1_)g sinθ+m_2_gl_2_sin(θ+φ)

MUSs=NETs+INTs+Gs

## Elbow

NETe＝{ m_2_l_2_^2^ +I_2_}$\ddot{\varphi}$

INTe={m_2_l_2_(l_2_+L_1_cosφ)+I_2_}$\ddot{\theta}$+(m_2_L_1_l_2_sinφ) $\dot{\theta}^{2}$

Ge=m_2_gl_2_sin(θ+φ)

MUSe=NETe+INTe+Ge

### Constants

m_1_: Mass of upper arm

m_2_: Mass of forearm and hand

L_1_: Length of upper arm

l_1_: Length from shoulder to COG of upper arm

l_2_: Length from elbow to COG of forearm and hand

I_1_: Moment of inertia of upper arm about COG

I_2_: Moment of inertia of forearm about COG

θ, $\dot{\theta}$, $\ddot{\theta}$: Shoulder angle, angular velocity, angular acceleration

φ, $\dot{\varphi}$, $\ddot{\varphi}$: Elbow angle, angular velocity, angular acceleration

g: Gravity
